# DSH_YOLO enables factory farming production: a method for detecting and grading the cumulative internode length of tomato seedlings

**DOI:** 10.3389/fpls.2026.1815103

**Published:** 2026-04-16

**Authors:** Xin Jin, Long Zhang, Mingyong Li, Yibo Hou, Zhitao He, Zhaohui Du, Kaikang Chen

**Affiliations:** 1 College of Agricultural Equipment Engineering, Henan University of Science and Technology, Luoyang, China; 2 Science & Technology Innovation Center for Completed Set Equipment, Longmen Laboratory, Luoyang, China; 3 Collaborative Innovation Center of Machinery Equipment Advanced Manufacturing of Henan Province, Luoyang, China; 4 Chinese Academy of Agricultural Mechanization Sciences Group Co., Ltd, Beijing, China

**Keywords:** deep learning, grading test, internode length, semantic segmentation, tomato seedlings

## Abstract

**Introduction:**

The industrial cultivation of tomato seedlings requires a high degree ofuniformity and consistency in grading. However, traditional grading methods based on phenotypictraits such as leaf area and canopy width are susceptible to environmental conditions, therebylimiting the accuracy and efficiency of grading. Since cumulative internode length is relativelystable and closely correlated with seedling vigor, this study aims to develop an accurate methodfor measuring and grading the cumulative internode length of tomato seedlings.

**Methods:**

A tomato seedling cumulative internode length detection method, termedDSH_YOLO (Deformable Convolution-SIoU-Haar Wavelet Downsampling YOLO), wasproposed based on the YOLOv8-seg framework. First, deformable convolution was introducedinto the backbone to enhance feature extraction for curved stems and occluded regions. Second,the original loss function was replaced with SIoU to improve the alignment between predictedregions and the actual stem structure. Third, a Haar wavelet downsampling module was embeddedinto the backbone to preserve high-frequency detail information and reduce information loss underocclusion conditions. An intelligent grading system was further developed to verify the practicalapplicability of the proposed method.

**Results:**

Experimental results showed that DSH_YOLO achieved a Precision of 96.1%, aRecall of 94.3%, and an mAP@0.5 of 92.1%. Compared with the baseline YOLOv8 model, theproposed method substantially improved segmentation performance for cumulative internoderegions in tomato seedlings. In prototype validation, the intelligent grading system achieved anaverage grading success rate of 87.50%, with an average cumulative internode length error of 8.0mm.

**Discussion:**

The results indicate that DSH_YOLO and the grading system can meet therequirements for large-scale grading and detection of tomato seedlings, demonstrating highdetection accuracy and success rates. This approach can provide insights for grading other types ofseedlings during their growth stages and offer support for seedling production and thedevelopment of intelligent agricultural equipment.

## 1 Introduction

In the tomato production stage, the growth of seedlings determines the quality of the plant (Markovic et al., 1996). Therefore, it is very important to accurately assess the growth level of tomato seedlings during the seedling stage. Cumulative internode length is one of the key factors determining plant height, and the internodes are responsible for transporting nutrients up and down the plant (Sun et al., 2019). Excessively long cumulative internode length indicates overly rapid vegetative growth, which reduces the number of leaves, impairs the accumulation of photosynthetic products, and often leads to leggy growth; conversely, excessively short cumulative internode length typically suggests that plant growth is inhibited. This metric can be assessed by observing the phenotypic characteristics of tomato seedlings; different phenotypic traits correspond to different growth stages of the seedlings and serve as an important basis for intuitively reflecting their growth vigor and health status (Pound et al., 2017).Precise grading of tomato seedlings is crucial for subsequent classification and cultivation management (Ali Ashraf et al., 2011), as it ensures consistent growth vigor within a single batch of seedlings and enables the industrial-scale, large-scale production of high-quality seedlings. Generally, the detection of the activity grading of tomato seedlings is mainly judged by observing characteristics such as leaf color (Chiu et al., 2006) and crown size (Zhu et al., 2023). However, these methods have significant limitations. Detection based on leaf characteristics is easily affected by environmental factors such as moisture, temperature, and light (Hott et al., 2018), which greatly reduces the accuracy and reliability of the detection results and makes it difficult to achieve batch and efficient evaluation.

As an important agronomic trait that influences plant structure and yield, extreme values in cumulative internode length often indicate environmental stress or genetic defects, requiring timely intervention (Sun et al., 2019). Sibomana et al. (2013) found in their study that cumulative internode length can serve as an indicator of stress factors such as drought and salinity. In agronomy, internode length refers to the distance between two consecutive nodes along a plant stem. As an important indicator of plant growth, the cumulative internode length of tomato seedlings is relatively stable and less susceptible to significant changes caused by short-term fluctuations in water, temperature, and humidity; therefore, it serves as a potential high-quality indicator for evaluating seedling grades. Consequently, accurately measuring the cumulative internode length of tomato seedlings is a key factor in their grading.

In terms of measuring cumulative internode length in plants, researchers in the field have already made some progress in this area. Yamamoto et al. (2016) employed an approach based on image analysis and machine learning to detect nodes and estimate internode lengths in tomato seedlings. This method consists of three steps: node detection, estimation of node order, and estimation of internode lengths. However, because this method relies on traditional machine learning, its recognition efficiency is not high. Boogaard et al. (2020) proposed a method for estimating cumulative internode length and tracking its development over time. This method also involves first detecting relevant nodes, then determining the order of each node, and finally estimating the cumulative internode length of the cucumber. The algorithm achieved precision and recall of 0.95 and 0.92, respectively. A few years later, Boogaard et al. (2023) proposed a method for estimating cumulative internode length from 3D point clouds of cucumber plants, achieving higher detection accuracy than previous studies. Hu et al. (2023) used an improved YOLOv5 model to detect nodes and estimate inter-node lengths in crops such as chili peppers. Although the study utilized deep learning to improve the speed of cumulative internode length identification, the process of first identifying nodes via object detection and then calculating the cumulative internode length reduced the accuracy of the nodes’precise coordinates. However, current research on automated detection of cumulative internode lengths using deep learning remains limited, and a mature technical framework has yet to be established.

With the rapid development of computer vision and deep learning, visual technology has provided powerful tools for detection tasks in the agricultural field (Jin et al., 2024; Kamilaris and Prenafeta-Boldú, 2018). Maraveas (2024) conducted a systematic review of the developments in plant phenotyping image analysis technology since 2020, noting that technologies such as artificial intelligence, machine learning, 2D/3D image reconstruction, and high-throughput image analysis are driving the evolution of plant phenotyping toward higher precision, automation, and intelligence. These technologies have already been widely applied in fields such as crop growth monitoring, trait extraction, and automated operations. In agricultural vision tasks, model selection depends not only on recognition accuracy but also on a comprehensive consideration of inference speed and practical deployment requirements. In their mobile-based research on freshness recognition for apples and lettuce, Maraveas et al, 2025 compared various deep learning models and ultimately selected a network architecture more suitable for practical deployment based on the comprehensive performance of accuracy, parameter count, and inference speed. For the task of detecting and grading the cumulative internode length of tomato seedlings studied in this paper, selecting the YOLO framework, which balances detection performance with real-time capabilities, is more reasonable. The YOLO (Redmon et al., 2016) series of algorithms has demonstrated outstanding performance in various object detection and segmentation tasks due to its efficiency, accuracy, and generalization ability. Badgujar et al, 2024 also pointed out in their recent review that the research and application of YOLO in agriculture is developing rapidly. Many studies have also demonstrated the effectiveness of the YOLO algorithm in agricultural detection. For example, Siregar et al, 2025 developed a disease detection system for tomato leaves based on YOLOv10, which improved the accuracy of disease identification. Mao et al. (2024) proposed a deep learning-based method for estimating the main stem length of sweet potato seedlings based on YOLOv5 and DeepLabv3, which effectively solved the problem of incomplete main stem features caused by leaf occlusion in the middle of sweet potato seedlings and improved the accuracy of estimating the main stem length of sweet potato seedlings. Wang et al. (2026) implemented real-time detection and segmentation of winter wheat weeds based on YOLOv10, which can be used for the precise identification and removal of weeds in the field. Xue et al. (2025) proposed a framework for the automatic segmentation and grading of tomato defects based on YOLOv11, which can adaptively capture subtle changes and blurred boundaries in tomato defects, making it significant for the accurate identification of surface defects in tomatoes. It is evident that deep learning-based segmentation models can play a significant role in detecting the cumulative internode length of tomato seedlings. Against this backdrop, the YOLOv8 model was selected as the base framework for this paper due to its balance of detection accuracy and inference efficiency.

To address the challenge of classifying tomato seedlings based on their cumulative internode length as an indicator of vitality, this paper proposes a deep learning-based system for detecting and classifying the cumulative internode length of tomato seedlings. A dataset of cumulative internode lengths was established by collecting images of tomato seedlings during their growth period. In terms of machine deployment, to improve the speed and accuracy of tomato seedling detection, a system for measuring cumulative internode length was integrated, comprising photoelectric sensors, an Aoni C96 camera, and a robotic arm for seedling handling. To address issues such as the diverse postures of tomato seedlings and the tendency for nodes to be obscured, we propose a multi-scale fusion-based network for detecting cumulative internode length in tomato seedlings: DSH_YOLO. Multiple deformable convolutions are introduced into the backbone to dynamically adjust the shape of the convolutional kernels to adapt to the curved characteristics of the seedling stems; the loss function is optimized to SIoU, and an angle penalty term is introduced to encourage the model to align the orientation of the main stem with the prediction bounding box; a Haar wavelet downsampling module is embedded into the CSPDarknet backbone to decompose the low-frequency and high-frequency components of the feature map, preserving high-frequency texture information of the stem edges and nodes. This study extends real-time cumulative internode detection technology from static analysis to dynamic robotic detection scenarios, addressing the challenge of acquiring stem morphological parameters and thereby providing a basis for seedling grading tasks. Unlike traditional methods that first detect tomato seedling nodes using object detection and then calculate the cumulative internode length ratio based on those nodes, the semantic segmentation algorithm proposed in this study directly generates a segmentation map of the cumulative internode lengths of tomato seedlings, effectively reducing the computational complexity of the algorithm.

## 2 Materials and methods

### 2.1 Materials

#### 2.1.1 Work background

**Figure 1:**
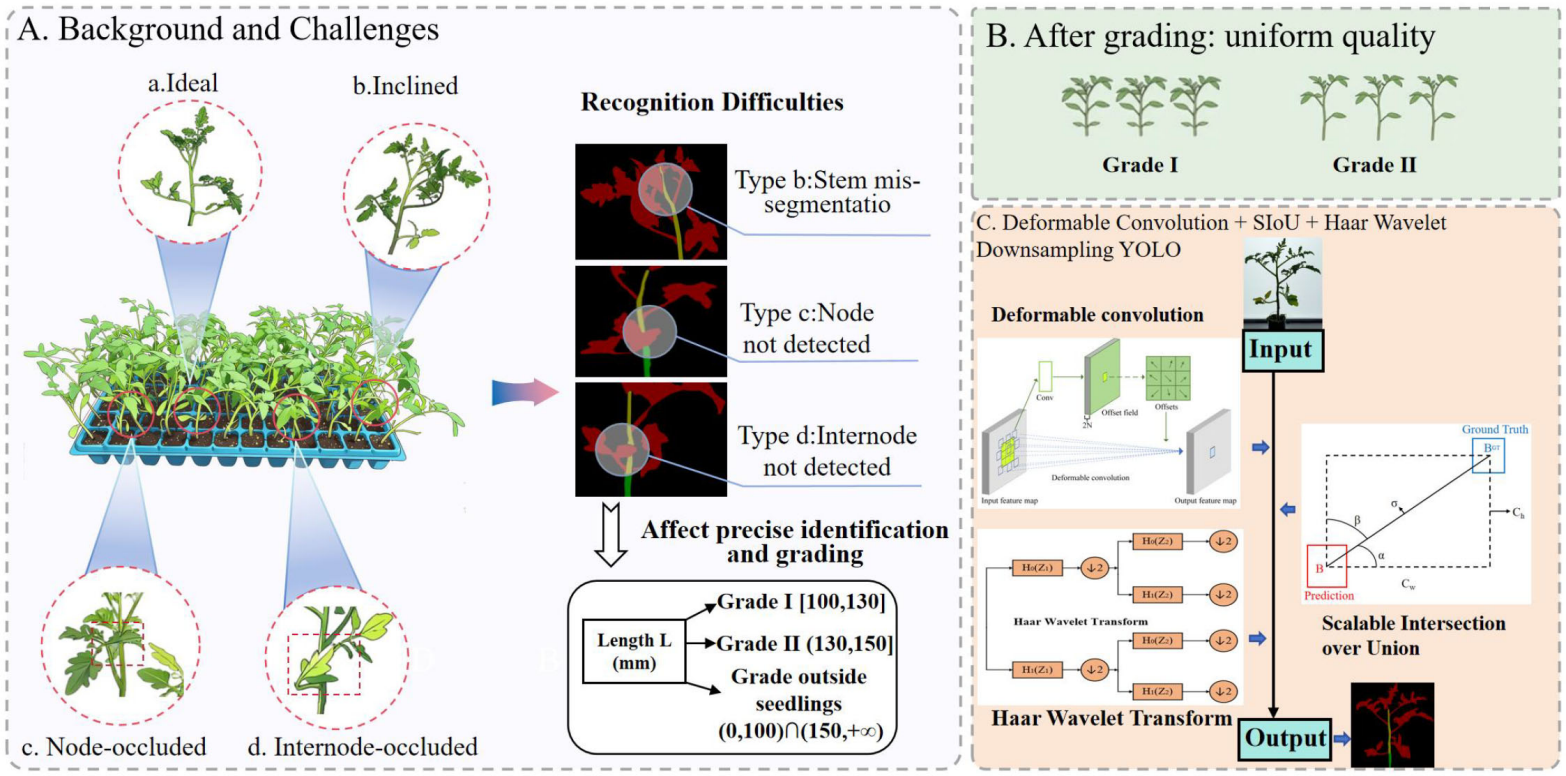
Working principle diagram. **(A)** Back and challenges. **(B)** After grading:uniform quality. **(C)** Deformable Convolution + SIoU + Haar Wavelet Downsampling YOLO.

During the seedling grading process, seedlings with uniform growth and clear, easily distinguishable features are more conducive to accurate identification. However, due to various factors such as the environment and genetic material, even seedlings grown in the same environment and batch often fail to achieve uniform growth. Common issues include curved branches and leaves, as well as obscured nodes and internodes, which directly impact the effectiveness of grading and subsequent fruit growth; Additionally, cumulative internode length is embedded within the main stem, requiring precise segmentation based on the similar characteristics of the main stem and branches. Furthermore, nodes—being minute features—must be effectively predicted and identified in cluttered and obstructed environments. These issues pose significant challenges to the task of grading seedlings based on cumulative internode length. The real challenges and technical difficulties mentioned above are visualized in **Figure 1**.

#### 2.1.2 Experiment materials

The tomato seedlings used in this study were of the Millennium variety. Due to the limited availability of public datasets for tomato seedlings, Millennium tomato seedlings cultivated at Chengyan Seedling Base in Luoyang City, Henan Province, were selected as the experimental variety to obtain more experimental data. All tomato seedlings used in the experiment were from the same batch, with the following cultivation parameters: seedling age 30–35 days, stem diameter 2.8-3.5 mm, number of main stem nodes 4-6, and 72-cell seedling trays with a size of 540 * 280 mm.

**Figure 2:**
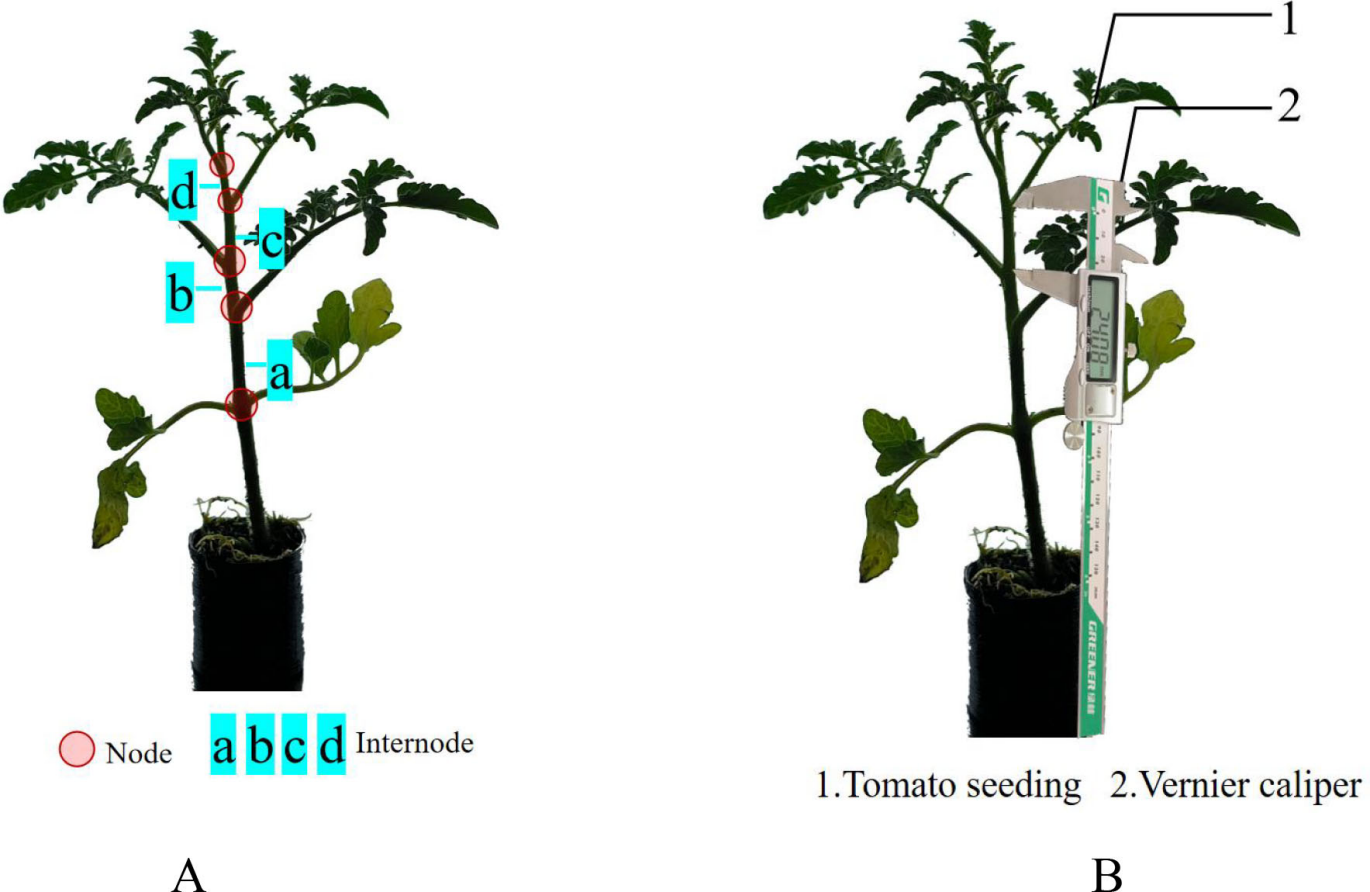
Procedure for measuring the cumulative internode length of tomato seedlings. **(A)** Diagram of tomato seedling nodes and internodes. **(B)** Measurement of cumulative internode length in tomato seedlings.

Traditional tomato seedling grading is mostly based on morphological characteristics such as plant height and crown width. However, these indicators are easily affected by environmental factors such as water and temperature, resulting in poor stability. For this reason, this study selected cumulative internode length—a more stable trait—as the criterion for grading tomato seedlings. Internode length in tomato seedlings refers to the distance between two adjacent nodes on the main stem. This study focuses on the sum of the lengths of all internodes, that is, the cumulative length of all internodes from the lowest cotyledon node to the highest growth point on the main stem, as shown by the sum of the lengths of four small internodes a-b-c-d in **Figure 2**.In this study, the cumulative span lengths were measured manually using a GREENER digital vernier caliper (accuracy: 0.01 mm) and recorded for comparison with the results obtained from the algorithm to assess measurement errors. The test site was the Facility Agriculture Robot Laboratory of the College of Agricultural Equipment Engineering, Henan University of Science and Technology, Luoyang City, Henan Province (112.37378E, 34.661055N).

When establishing the classification of cumulative internode length for the Millennium tomato variety grown in greenhouses, we consulted relevant agronomic experts. Based on data distribution and biological significance, we adopted a non-equidistant classification to ensure that each grade is distinct and aligns with actual growth conditions. The classification criteria for cumulative internode length of the tomato seedlings studied in this paper are defined as shown in **Table 1**. Specifically, Grade I and Grade II seedlings are primarily used for management strategies such as planting within the same batch or selling based on quality; seedlings outside these grades are considered inferior due to excessive growth or stunted growth.

**Table 1:** Grading standards for tomato seedlings.

Cumulative internode length (mm)	Grade
[100,130]	I
(130,150]	II
(0,100)∩(150,+∞)	Grade outside seedlings

#### 2.1.3 Dataset construction

**Figure 3:**
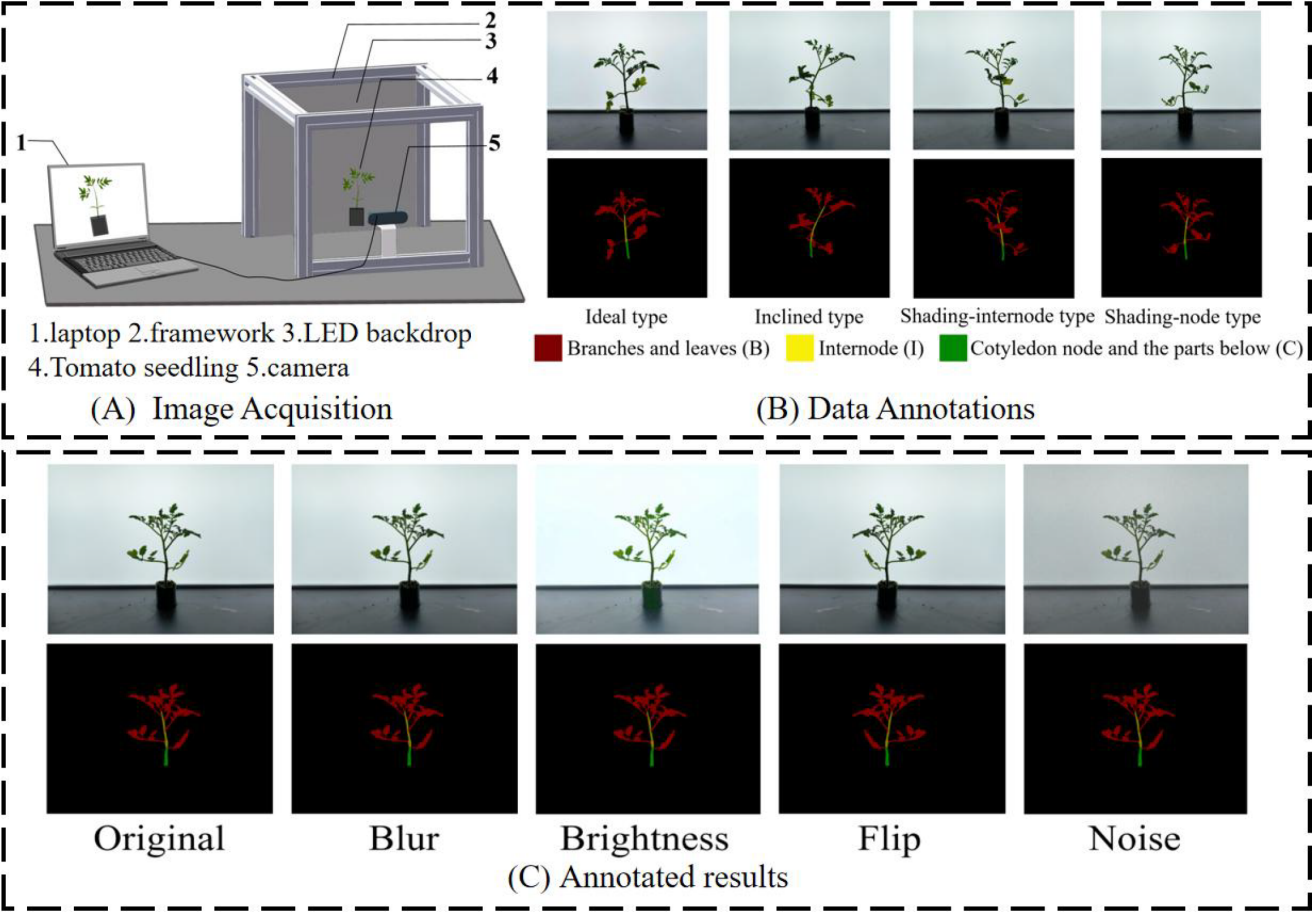
Data set processing process. **(A)** Image acquisition setup. **(B)** Data annotations. **(C)** Annotated results.

The camera model used in the research is Aoni C96, with an image acquisition resolution of 3840*2160 pixel. During data acquisition, in order to closely approximate the real environment after algorithm deployment, a simulated environment was set up in advance as shown in **Figure 3A**. A total of 1,286 valid images were collected, of which 1,100 were used for subsequent data augmentation and model training, while 186 were retained for algorithm validation and to compare the error in cumulative internode lengths with manual measurements. After obtaining the raw images captured by the camera, import the photos into the labelme image annotation tool for polygon annotation. Set the labels for the branches and leaves section of the tomato seedlings to 0, the cumulative internode length section to 1, and the cotyledon node and the parts below section to 2. When the internodes are occluded, only the visible real parts in the image are annotated. The data annotation results are shown in **Figure 3B**.

The training of deep learning models is highly dependent on large-scale and diverse data, while the image data obtained in real-world scenarios is often limited and difficult to meet the complex learning needs of the model. To this end, this paper uses operations such as RandomBrightness, GaussNoise and GaussianBlur to augment the collected images to avoid overfitting and excessive test randomness that may occur during the testing process (Wei et al., 2025), as shown in **Figure 3C**.RandomBrightness simulates images taken under different lighting conditions by varying the brightness of the image. The brightness variation range is set to ±20% to simulate natural lighting changes while maintaining clear intersegmental features, thus improving the model’s robustness to changes in light intensity. GaussNoise introduces random noise into the image, allowing the model to learn image features with noise interference. The standard deviation is set to 5-25, striking a balance between introducing realistic noise and preserving details, improving the model’s ability to recognize noisy images. GaussianBlur blurs the image, simulating the effect of images being shot in scenarios such as out-of-focus conditions. It uses a 3×3 kernel size with a standard deviation range of 0.1-2.0, ensuring that the model’s adaptability to blurred images is enhanced without affecting intersegmental edge accuracy, and expanding the range of features the model can learn. The above data augmentation operations effectively increased the diversity of the data, enabling the model to be exposed to a variety of features during training, thereby greatly improving the model’s generalization ability and accuracy in different scenarios. The aforementioned data augmentation techniques effectively increased data diversity, enabling the model to be exposed to a wide range of features during training. This significantly improved the model’s generalization capabilities across different scenarios and enhanced its accuracy in practical applications. By randomly applying two of the four techniques to each dataset, the dataset size was tripled after augmentation, resulting in a total of 3,300 samples.

### 2.2 Methods

**Figure 4:**
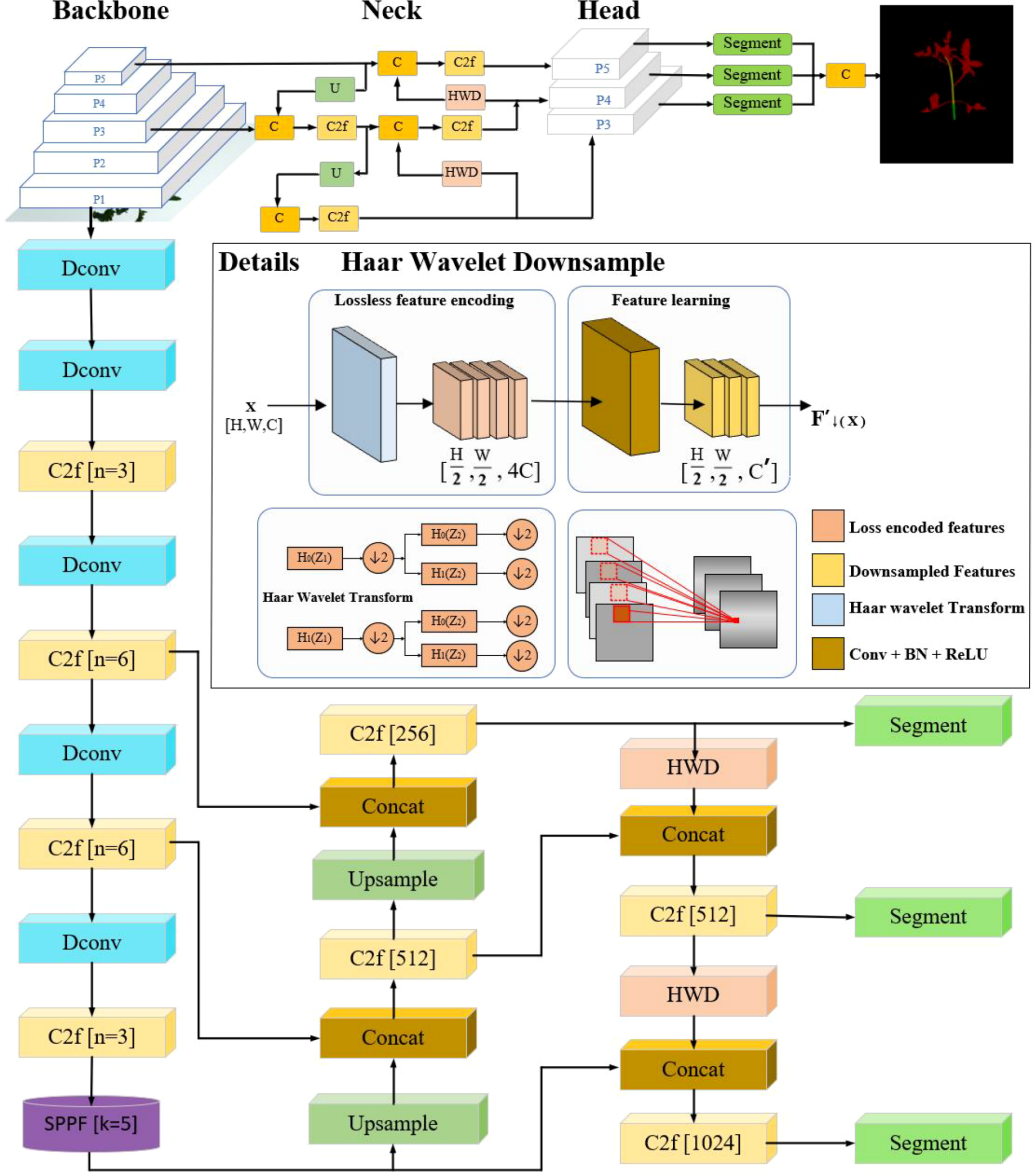
DSH_YOLO network overall framework.

To solve the problem of nodes and internodes being easily obscured and difficult to identify when detecting the cumulative internode length of tomato seedlings, this paper proposes a multi-scale fusion based tomato seedling cumulative internode length detection network, DSH_YOLO, for detecting the cumulative internode length of tomato seedlings. On the basis of maintaining the overall framework of YOLOv8-seg unchanged, this network has made three main improvements: firstly, multiple deformable convolution (DConv) layers are introduced into the YOLOv8 backbone network, and the feature extraction ability of curved stems and non rigid deformation areas is enhanced by adaptively adjusting the sampling position; Secondly, the bounding box regression loss is replaced from the original CIoU to SIoU, and angle cost is introduced to make the model more focused on aligning the main stem with the predicted box; Finally, a Haar wavelet downsampling (HWD) module is embedded in the CSPDarknet backbone to decompose the feature map through low-frequency (A) and high-frequency (H/V/D) components, preserving the high-frequency texture information of stem edges and nodes, thereby reducing the information loss caused by traditional pooling operations. Except for the above improvements, the rest of the network structure remains consistent with YOLOv8-seg. The DSH_YOLO algorithm framework is shown in **Figure 4**.

#### 2.2.1 Deformable convolution module

The complex structure of tomato seedlings makes it difficult to identify seedling phenotypic traits (Solimani et al., 2024), and the nodes of tomato seedlings are easily obscured by irregularly growing branches, leaves, etc. The kernel of traditional convolution is fixed, which is difficult to adapt to the bending shape of tomato seedling stems and the non-rigid deformation of internode structure. At the same time, it is impossible to accurately obtain information of the obscured area because the sampling position is fixed. Therefore, this paper introduces deformable convolution (Dai et al., 2017). Deformable convolution is an improved convolution operation that can dynamically learn the offset so that the sampling point can be adaptively adjusted according to the target deformation.

The traditional convolution structure expression is shown in Equation 1:

(1) $$y(p_{0})=\sum_{p_{n}\in R}w(p_{n})x(p_{0}+p_{n})$$

Where ***y(****p*_0_***)*** represents the value of the output feature map at position *p*_0_, R denotes the set of regular sampling positions of the convolutional kernel, *w*(*p_n_*) denotes the convolutional weight corresponding to the nth sampling position, and x denotes the pixel value of the input feature map.

Deformable convolution, based on Equation 1 above, introduces an offset for each point. This offset is generated by the input feature map and another convolution, as shown in Equation 2:

(2) $$y(p_{0})=\sum_{p_{n}\in R}w(p_{n})x(p_{0}+p_{n}+\Delta p_{n})$$

Where ***Δ****p_n_* is the learnable offset of the nth sampling point, which is predicted by an additional convolutional layer.

**Figure 5:**
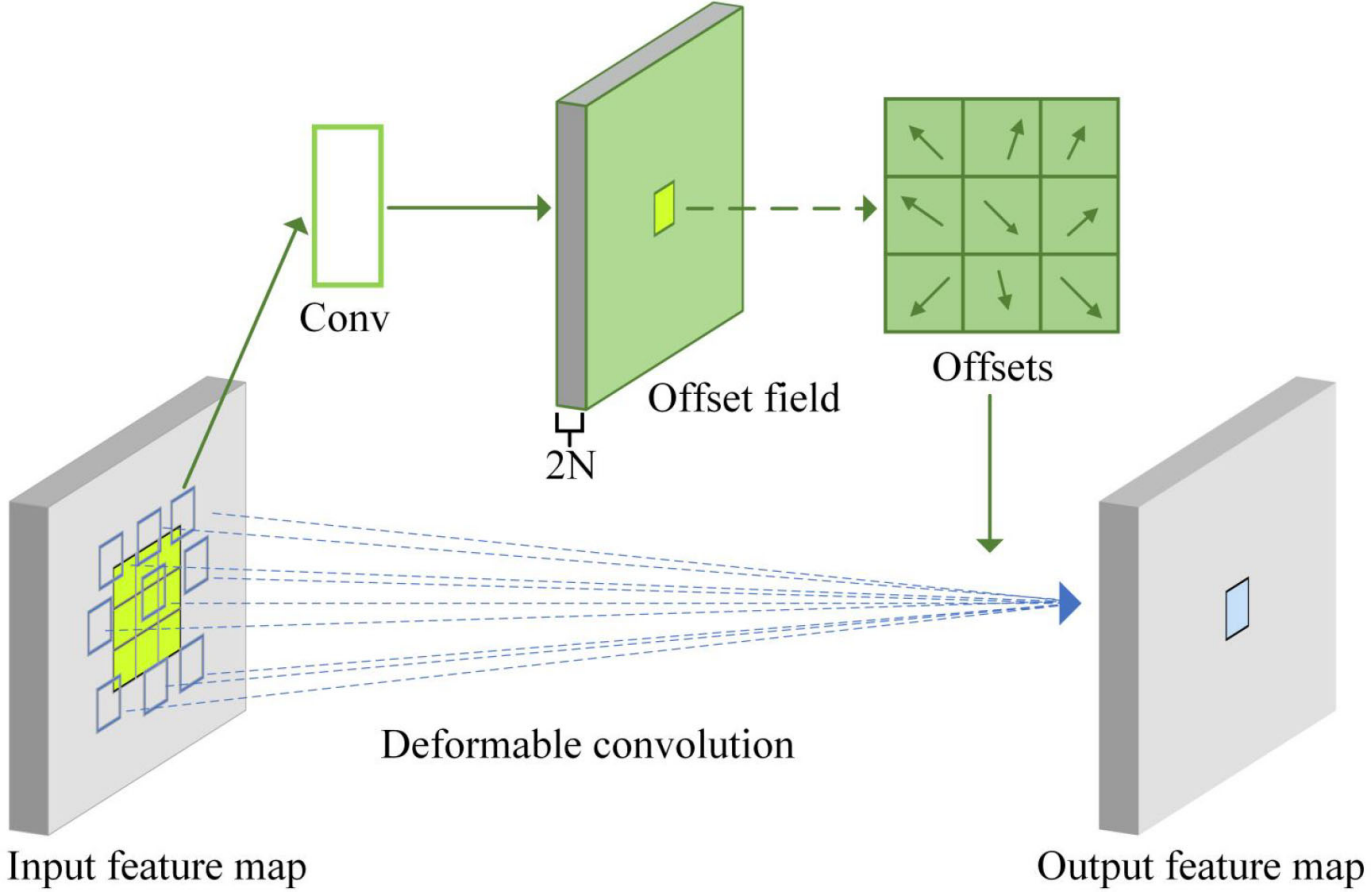
Schematic diagram of deformable convolution.

**Figure 5** illustrates a deformable convolution. As shown, offsets are generated by an additional convolutional layer with a channel dimension of 2N. The 2 corresponds to the X and Y 2D offsets, and N is determined by the kernel size (e.g., a 3*3 kernel corresponds to N = 9). Compared to the regular grid sampling mode of conventional convolution (shown as green rectangles in **Figure 5**), deformable convolution implements an adaptive sampling mode based on input feature content (marked by blue rectangles in **Figure 5**). Specifically, this architecture includes two parallel feature processing paths: the upper path predicts offsets through convolutional layers, and the lower path performs feature aggregation based on dynamically sampled coordinates to complete the deformable convolution operation.

**Figure 6:**
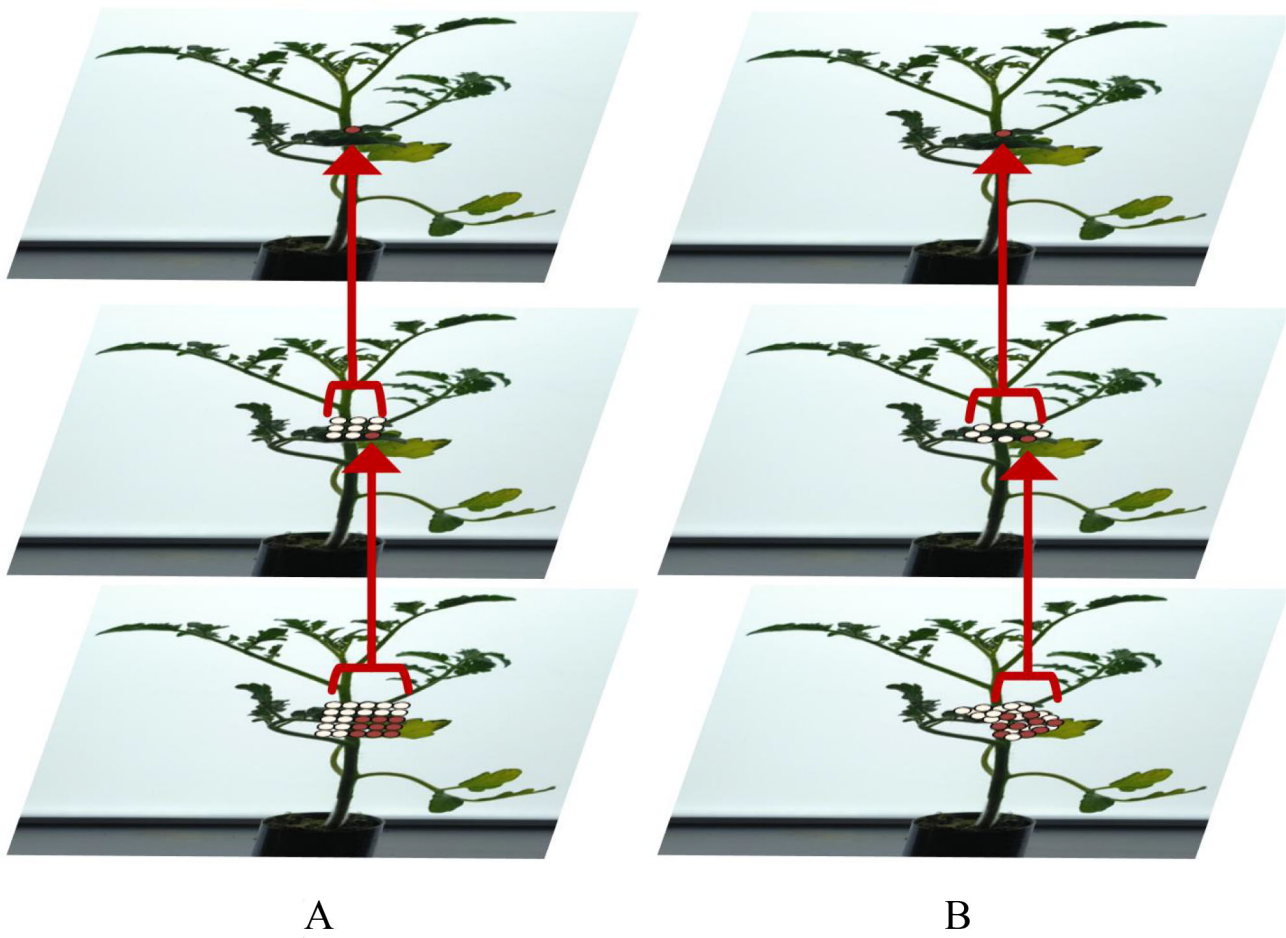
Comparison of different convolutions. **(A)** Standard Convolution. **(B)** Deformable Convolution.

At the junction of the stem and leaves of tomato seedlings, the offset can guide the convolution kernel to focus on key areas. The sampling position is more in line with the shape and size of the tomato seedlings, rather than being forcibly aligned to a fixed network. Compared with the original convolution, it can improve the boundary accuracy of network segmentation. The sampling of the convolution kernel can be adjusted according to the features of the visible part and the overall shape of the tomato seedling to obtain as much relevant information as possible around the area covered by leaves, as shown in **Figure 6**.

#### 2.2.2 SIoU loss function module

Due to the slender structure and disproportionate growth of tomato seedlings, the original CIoU loss function of the YOLOv8 network—which is primarily designed for object detection and general image segmentation tasks—suffers from issues such as directional insensitivity and size bias. These issues cause bounding box regression to fluctuate due to the multi-directional growth characteristics of tomato seedlings’ branches and leaves, making it difficult to directly meet the requirements for high-precision measurement of cumulative internode length. Therefore, this study introduces the bounding box loss function SIoU (Gevorgyan, 2022) to enhance the model’s sensitivity to internode region features by optimizing the overlap between the predicted box and the actual region, thereby improving the detection accuracy of internode region.

**Figure 7:**
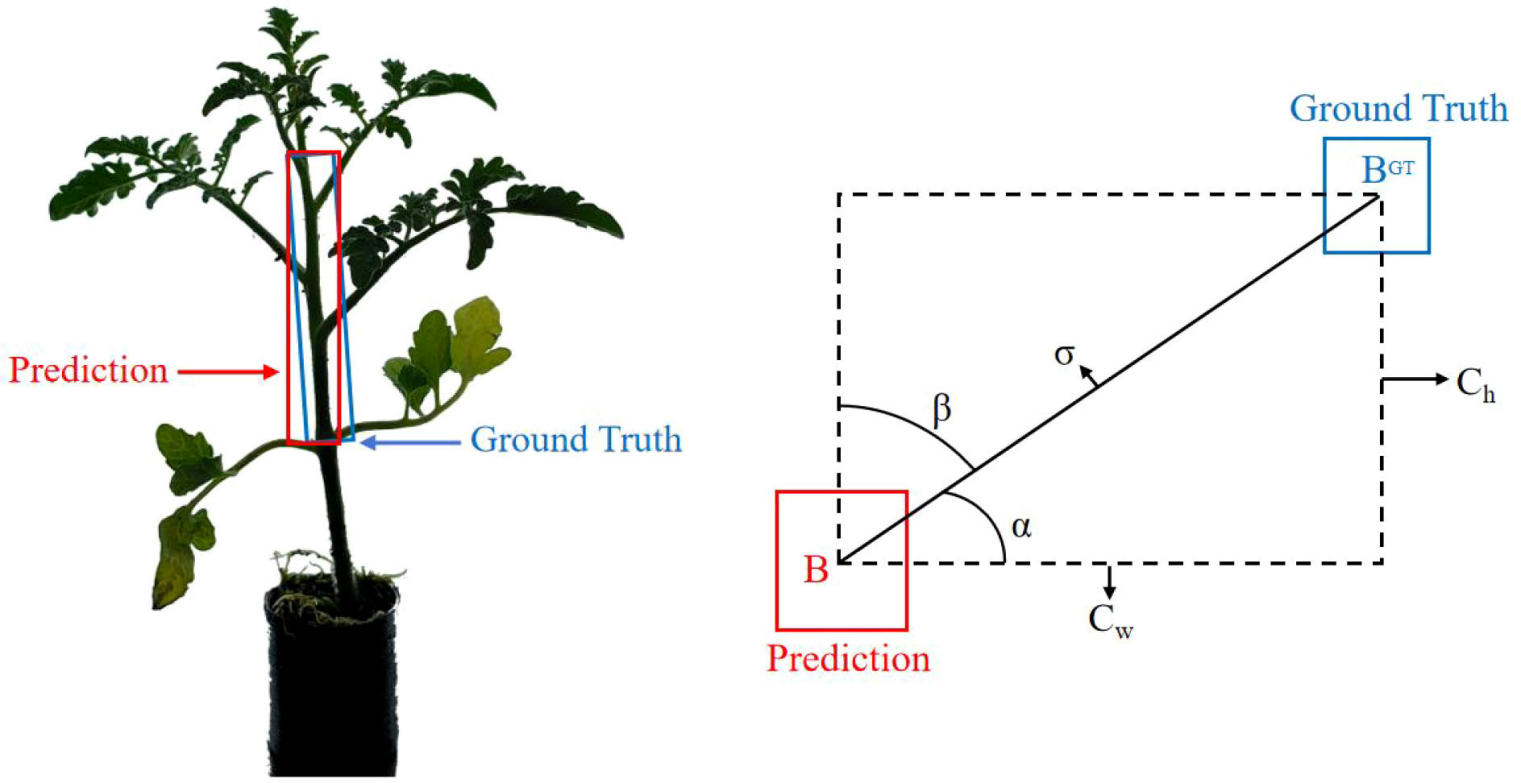
Schematic diagram of SIoU loss function.

SIoU further considers the vector angle between the ground truth bounding box and the predicted bounding box, redefining four loss functions. Considering the multi-directional growth of branches and leaves in the actual tomato seedlings of this study, and the slender internode structure, the Angle Cost of SIoU is emphasized to effectively constrain the directional alignment of the segmentation boundaries and reduce missegmentation in the branch-leaf intersection area. The angle loss, by introducing a vector angle penalty mechanism, addresses the insensitivity of the traditional CIoU loss function to the bounding box orientation. **Figure 7** shows a schematic diagram of the SIoU loss function, and Equation 3 is the formula for calculating the angle loss:

(3) $$\Lambda =1-2*sin2(arcsin(\frac{c_{h}}{\sigma })-\frac{\pi }{4})$$

Where *c_h_* is the vertical distance between the center points of the predicted bounding box and the ground truth bounding box, σ is the Euclidean distance between the center points of the predicted bounding box and the ground truth bounding box, and α is the angle between the line connecting the centers of the predicted bounding box and the ground truth bounding box and the coordinate axis.

In practical terms, the Λ penalty is minimized when the angle α between the stem axis and the coordinate axis approaches 0 or 90°, at which point the predicted bounding box tends to align with the actual stem axis. Conversely, the Λ penalty is maximized when α approaches 45°, forcing the model to prioritize correcting directional deviations and preventing the network from generating multiple short bounding boxes along curved paths. Especially when the angle between the lateral branches and the main stem is close to 45°, the Λ penalty can suppress erroneous direction regression, ensuring accurate internode segmentation of the main stem. The stem tilt of tomato seedlings can cause internode segmentation bounding boxes to shift. The angle loss, by introducing axial direction constraints, allows the predicted bounding box to converge quickly to the actual stem axis, reducing segmentation errors caused by stem bending.

#### 2.2.3 Haar Wavelet downsampling module

The YOLOv8 network uses Streided Convolution and MaxPooling by default for feature map downsampling, which can expand the receptive field and enhance the model’s ability to capture features at different scales. However, this method has many limitations when it comes to segmenting the complex morphology of tomato plants. For example, pooling operations can blur weak edge regions at the internodes and where branches and leaves are obscured, leading to broken segmentation boundaries. High-frequency noise such as leaf textures can also easily mix with the main stem features in traditional downsampling, increasing the risk of missegmentation.

To address the shortcomings of the original downsampling method, this study introduces Haar Wavelet Downsampling (HWD) (Xu et al., 2023). The core idea of the HWD module is to use Haar Wavelet Transform to reduce the spatial resolution of the feature map while ensuring that all information is preserved. During the processing, this module encodes some spatial information into the channel dimension, making the feature expression richer and more effective.

**Figure 8:**
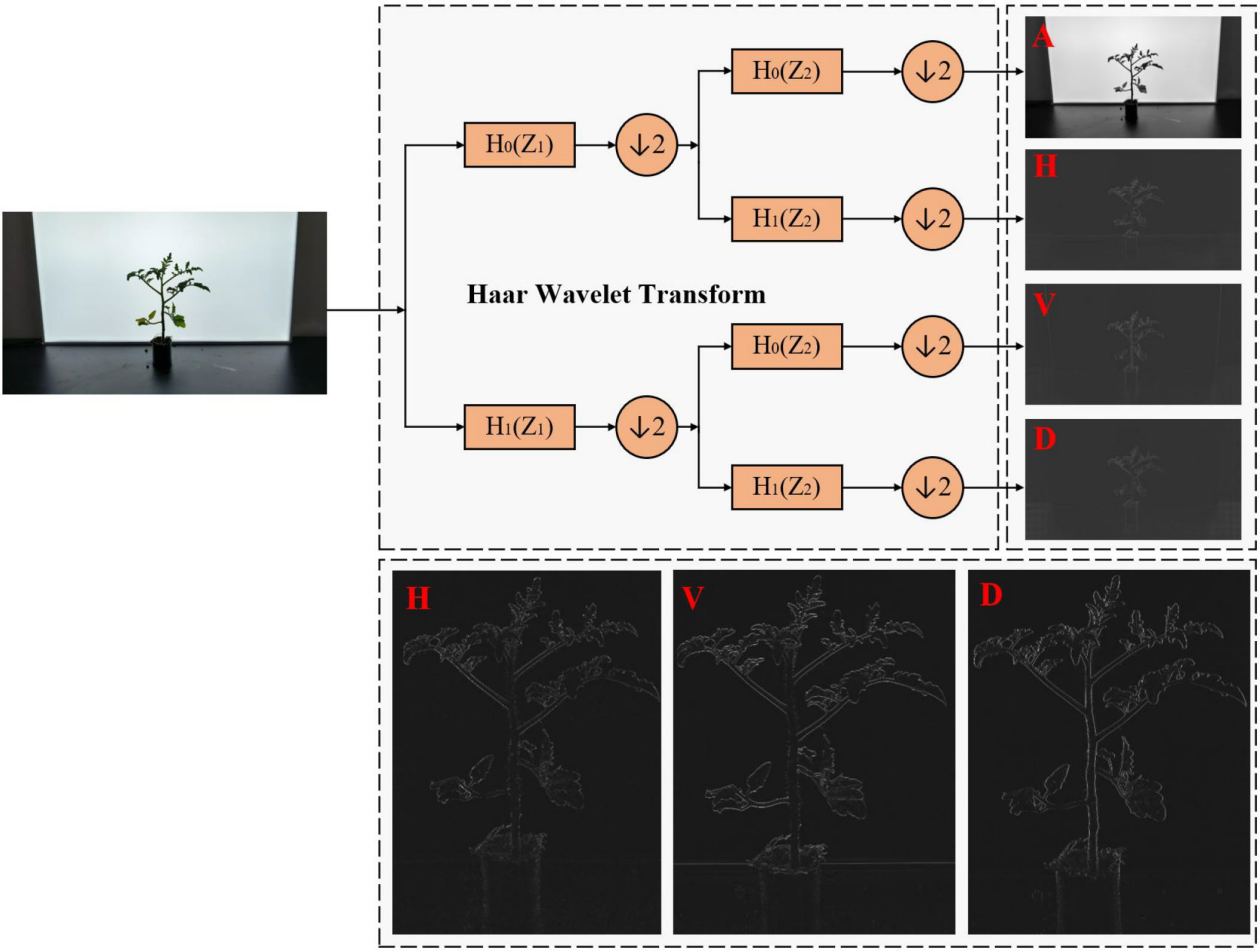
Haar Wavelet Transform structure diagram. **(A)** Approximation component. **(H)** Horizontal detail component. **(V)** Vertical detail component. **(D)** Diagonal detail component.

Specifically, in the context of this research, HWD decomposes the feature map into low-frequency approximation components (A) and high-frequency detail components (H, V, D) using Haar Wavelet Transform. These components represent the multi-scale spatial information of the tomato seedling image, providing a comprehensive and detailed description of the image features. While reducing resolution, it preserves the continuity of the stem bending path and the local features of the occluded area. This characteristic is crucial for accurately segmenting the complex morphology of tomato plants, especially in scenarios with bent stems and occlusion by branches and leaves, effectively improving segmentation accuracy. Introducing Haar wavelet downsampling into the object detection framework, through multi-directional feature decomposition and information entropy optimization, effectively solves the bottleneck of detail loss in traditional methods under curved stems and occlusion scenarios. The Haar Wavelet Transform structure is shown in **Figure 8**.

As shown in **Figure 8** above, the low-frequency component (A) is filtered using 2D-HWT to preserve the global structural features of the tomato seedling stem. The high-pass components (H, V, D) capture the edge gradients at the junctions of internodes and branches, as well as the overlapping contours of internodes and branches. Adaptive thresholding and weighted fusion are applied to the H, V, and D components to enhance the weak edge effect at the junctions of internodes and cotyledons and suppress leaf noise interference.

## 3 Experimental results and analysis

### 3.1 Experimental evaluation indicators

To objectively evaluate the segmentation performance of this algorithm on tomato seedlings and to verify the improvement effects of the DConv module, SIoU angle loss module, and HWD module in DSH_YOLO on the algorithm, we conducted ablation experiments on this algorithm, using precision (P), recall (R), and mean average precision (mAP@0.5) as evaluation metrics. Here, P represents the proportion of correctly segmented target regions out of all predicted positive regions, R represents the proportion of correctly segmented target regions out of all actual positive regions, and mAP@0.5 represents the mean average precision of all classes when the Intersection over Union (IoU) threshold is 0.5. A larger mAP@0.5 value indicates higher model recognition accuracy. The calculation formula is shown in Equations 4-7:

(4) $$P=\frac{TP}{TP+FP}$$

(5) $$R=\frac{TP}{TP+FN}$$

(6) $$AP=\int_{0}^{1}P(R)dR$$

(7) $$mAP=\frac{1}{N}\sum_{i=1}^{N}AP_{i}$$

Where TP (True Positive) represents the number of correctly segmented pixels, FP (False Positive) represents the number of pixels that were mistakenly identified as the target region, FN (False Negative) represents the number of pixels that were missed in the detection of the true target region, and N represents the total number.

### 3.2 Experimental conditions

All experiments in this paper were conducted under the same experimental conditions. The specific hardware and software configurations are as follows: the computer system was Windows 11, the GPU was an NVIDIA GeForce RTX 3050 with 12GB of video memory, Python 3.11 was used as the programming language for model building, and PyTorch version 2.3.1 was used as the deep learning framework. The model training hyperparameters were set as follows: a Stochastic Gradient Descent optimizer with a momentum of 0.937 was used, the initial learning rate was set to 0.01, and a Cosine Annealing strategy was used to dynamically adjust the learning rate. Training lasted for 300 epochs, with each training batch size of 16. To prevent overfitting during training, an Early Stopping strategy was adopted; training was terminated early if there was no improvement after 100 consecutive epochs of training.

### 3.3 Analysis of algorithm detection results

**Figure 9:**
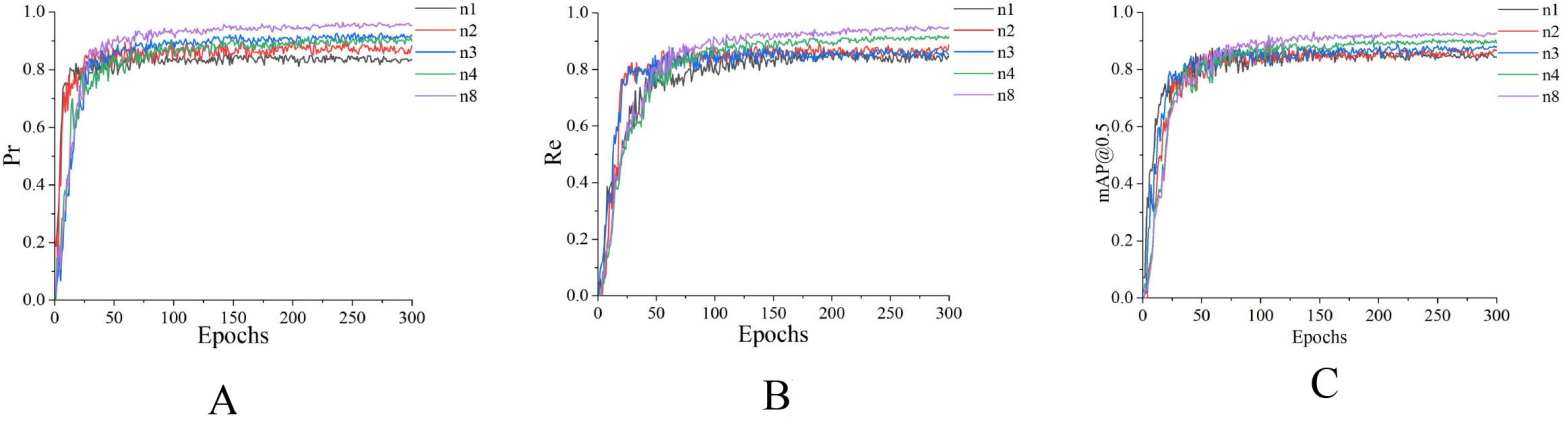
Comparison of module tests. **(A)** Accuracy comparison chart. **(B)** Recall rate comparison chart. **(C)** mAP@0.5 comparison chart.

To verify the optimization effect of the three improvement strategies mentioned above on the model, we conducted ablation experiments on the algorithm. The experimental results are shown in **Table 2** and **Figure 9**:

**Table 2:** Test data of each module.

Number	DConv	SIoU	HWD	Pr(%)	Re(%)	mAP@0.5(%)
n1				84.4	83.3	86.9
n2	✓			87.8	86.3	87.4
n3		✓		91.3	85.7	88.1
n4			✓	90.1	88.5	88.6
n5	✓	✓		91.6	89.2	88.5
n6	✓		✓	93.1	92.1	90.2
n7		✓	✓	92.5	91.5	90.9
n8	✓	✓	✓	96.1	94.3	92.1

In the table above, n1 refers to the original, unmodified YOLOv8 algorithm; n2 refers to the addition of the DConv module alone; n3 refers to the addition of the SIoU module alone; n4 refers to the addition of the HWD module alone; and so on, with n8 (DSH_YOLO) indicating the addition of all three enhancement modules to the YOLOv8 algorithm.

To avoid clutter caused by excessive lines in **Figure 9**, which could hinder observation, only the data for n1, n2, n3, n4, and n8 are visualized here. As shown in the experimental data in **Table 2** and **Figure 9**, when performing recognition after training the original network (n1), the recognition accuracy for the internodal sections of tomato seedlings was poor, with varying degrees of missed detections; when the DConv module was introduced independently (n2), precision increased by 3.4%, recall rose by 3%, and mAP@0.5 increased by 0.5%. This improvement is attributed to DConv’s adaptive receptive field, which enhances the network’s adaptability to the curved stems of tomato seedlings, thereby reducing false positives and false negatives; After introducing SIoU alone (n3), P, R, and mAP@0.5 improved by 6.9%, 2.4%, and 1.2%, respectively, compared to the baseline network. This is attributed to the angle penalty term in SIoU, which forces bounding boxes to align with the axial growth direction of the tomato seedling stems, thereby reducing directional deviations in predicted boxes caused by branch and leaf forking; After introducing the HWD module alone (n4), P, R, and mAP@0.5 improved by 5.7%, 5.2%, and 1.7% respectively compared to the baseline. Analysis indicates that HWD enhances high-frequency signals along the stem edges using Haar wavelets, improving the sensitivity of identifying thin and weak stem segments, while multi-scale frequency domain fusion alleviates feature confusion caused by overlapping branches, leaves, and internodes. When the DConv, SIoU, and HWD modules were simultaneously incorporated into the network (n8), P, R, and mAP@0.5 improved by 11.7%, 11%, and 5.2% respectively compared to the baseline. Experimental data also demonstrated that the three improved modules are technically complementary in this study.

**Figure 10:**
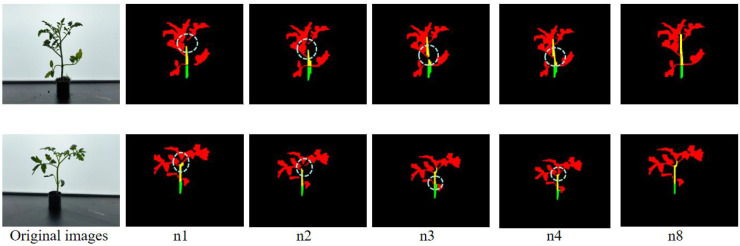
Comparison of recognition of different modules.

To visually verify the performance improvements of each module, we selected two images from an untrained dataset for a visual comparison. The results are shown in **Figure 10** (the edges of the seedlings were cropped to clearly highlight the differences in recognition). The gray circles in the figure indicate areas where the network failed to segment the tomato seedlings or did not segment them accurately, serving as a comparison with the DSH_YOLO detection results.

**Figure 11:**
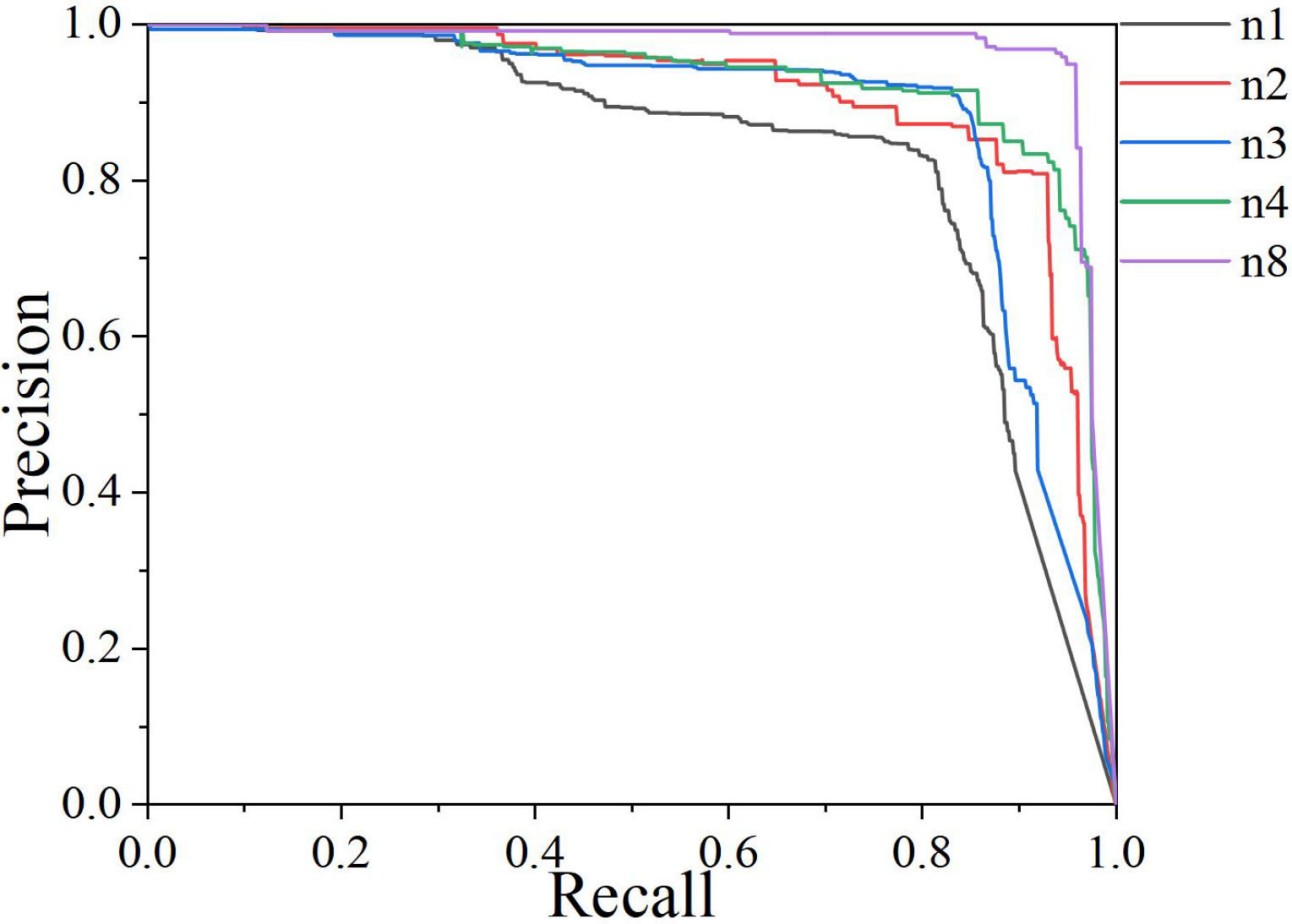
P-R curve comparison of different modules.

To further verify the performance of our algorithm, we plotted the PR curves after improving different modules, as shown in **Figure 11**. It can be seen that as the Recall value increases, the Precision of each module decreases to varying degrees. When Recall is less than 0.8, all modules maintain high Precision. However, when Recall is greater than 0.8, especially in the 0.9~0.95 range, our proposed algorithm DSH_YOLO still maintains high Precision and outperforms the other three modules. This indicates that DSH_YOLO can detect as many real targets as possible while ensuring the accuracy of the results, demonstrating that DSH_YOLO has superior overall performance and better robustness and generalization ability.

**Figure 12:**
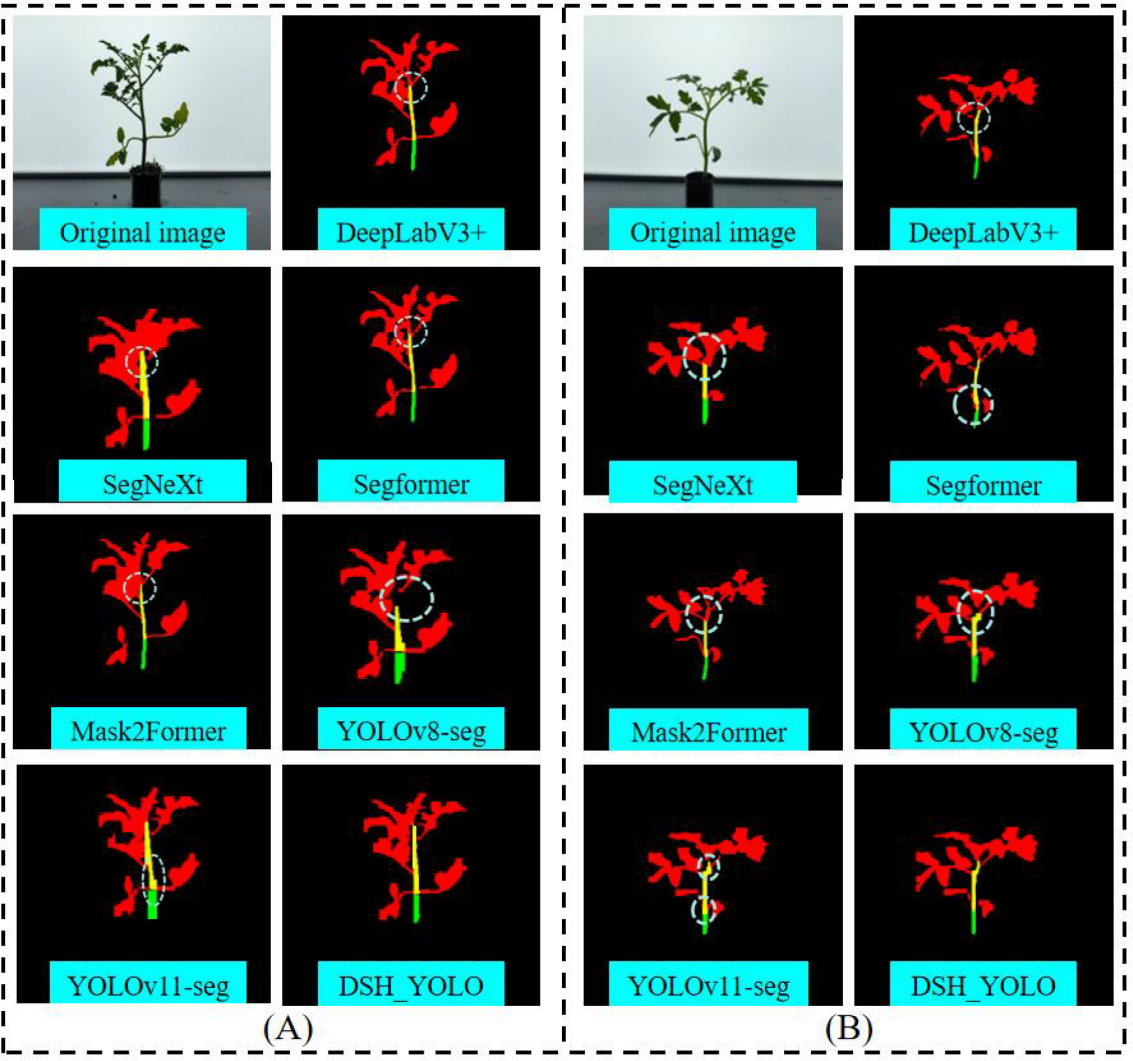
Comparison of recognition results of different models. **(A)** Example 1. **(B)** Example 2.

In addition, we conducted a comparative evaluation of the DSH_YOLO model proposed in this paper against current mainstream semantic segmentation algorithms, using Precision, Recall, and FPS as evaluation metrics. The results of this comparison are shown in **Table 3**. As can be seen from the data in the table, DSH_YOLO achieves the highest Precision, Recall, and FPS among the compared models. Compared to DeepLabV3+ (Chen et al., 2018), SegFormer (Xie et al., 2021), Mask2Former (Cheng et al., 2022), SegNeXt (Guo et al., 2022), YOLOv8-seg, and YOLOv11-seg, DSH_YOLO achieved 12.7%, 14.3%, 10.6%, 10%, 11.7%, and 6.6% higher Precision, respectively, Recall is 6.1%, 14.2%, 7.6%, 7.9%, 11%, and 3.9% higher, respectively, and FPS is 7, 8, 13, 6, 8, and 5 frames per second higher. Additionally, as shown in the comparison of different models on different images in **Figure 12**, the accuracy of image segmentation achieved by the proposed model in this paper is superior to that of the other compared models (shortcomings in the model’s segmentation are circled in **Figure 12**). These results demonstrate the accuracy and superiority of the DSH_YOLO model proposed in this paper for tomato seedling image segmentation, which can meet the requirements for detecting the cumulative internode length of tomato seedlings.

**Table 3:** Performance comparison of different models.

Model	Pr(%)	Re(%)	FPS
DeepLabV3+	83.4	88.2	20
Segformer	81.8	80.2	19
Mask2Former	85.5	86.7	14
SegNeXt	86.1	86.4	21
YOLOv8-seg	84.4	83.3	19
YOLOv11-seg	89.5	90.4	22
DSH_YOLO	96.1	94.3	27

**Figure 13:**
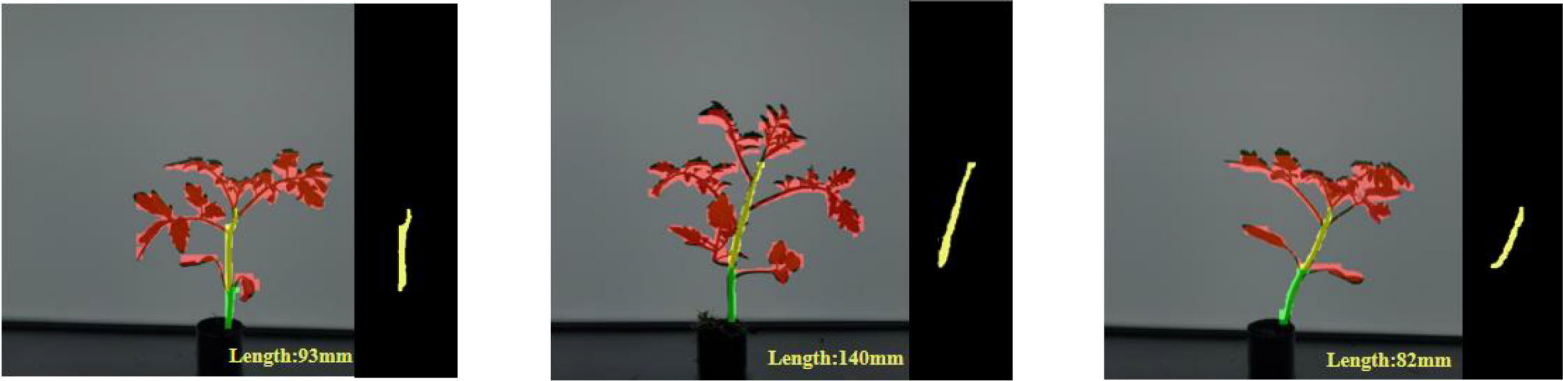
Recognition results.

To evaluate the reliability of the DSH_YOLO algorithm in this paper for measuring the actual length of the cumulative internodes of tomato seedlings, we first calibrated the camera using a pinhole imaging model. Using a checkerboard calibration board and Zhang Zhengyou’s calibration method, we obtained the camera’s intrinsic parameter matrix and distortion coefficients. We then used the obtained distortion coefficients to perform distortion correction on the original input image to eliminate geometric errors caused by lens distortion and ensure the geometric linearity of subsequent coordinate transformations. The trained DSH_YOLO algorithm is exported in ONNX format and accelerated for inference using the TensorRT engine on the NVIDIA Jetson edge computing platform. The system reads the distortion-corrected seedling images and based on the output internode region mask, applies the Zhang-Suen thinning algorithm to extract the centerline of the internode region. First, the segmentation results are binarized. After obtaining the internode region mask, isolated noise pixels are removed through connected component filtering and morphological denoising, retaining the main internode regions; Next, the binary mask is subjected to iterative thinning using the Zhang-Suen thinning algorithm to obtain a single-pixel-wide skeleton. This skeleton effectively preserves the topological structure and centerline information of the internode regions, enabling the reconstruction of the actual growth trajectory of the cumulative internode length in tomato seedlings. The recognition results are shown in **Figure 13**.

Let the ordered sequence of pixels on the skeleton be as shown in Equation 8:

(8) $$P=\{p_{1},p_{2},\ldots p_{n}\},p_{i}=(x_{i},y_{i})$$

Where *p_1_* and *p_n_* denote the two endpoints of the segmental skeleton, respectively, the arc length of the centerline of this segment in the pixel coordinate system can be expressed as in Equation 9:

(9) $$L_{px}=\sum_{i=1}^{n-1}\|p_{i+1}-p_{i}{\|}_{2}=\sum_{i=1}^{n-1}\sqrt{{\left(x_{i+1}-x_{i}\right)}^{2}+{\left(y_{i+1}-y_{i}\right)}^{2}}$$

Based on the longitudinal factor k (unit: mm/pixel) obtained from camera calibration, the actual cumulative span length L is given by Equation 10:

(10) $$L=k*L_{px}$$

### 3.4 Prototype experiment

The deep learning-based algorithm for detecting the cumulative internode length of tomato seedlings will be deployed and applied to a grading robot. The device mainly consists of a conveyor belt, a robotic arm module, a control cabinet, a vision monitoring system, and an LED supplementary lighting device. The main parameters are shown in **Table 4**.

**Table 4:** Main equipment parameters.

Device	Parameter
Camera model	Aoni C96
Sensor Model	PDR-Y35P30NS-Y
Image Acquisition Resolution	3840*2160 pixel
LED Light Intensity	100 Lux
Operating Air Pressure	0.4 MPa

**Figure 14:**
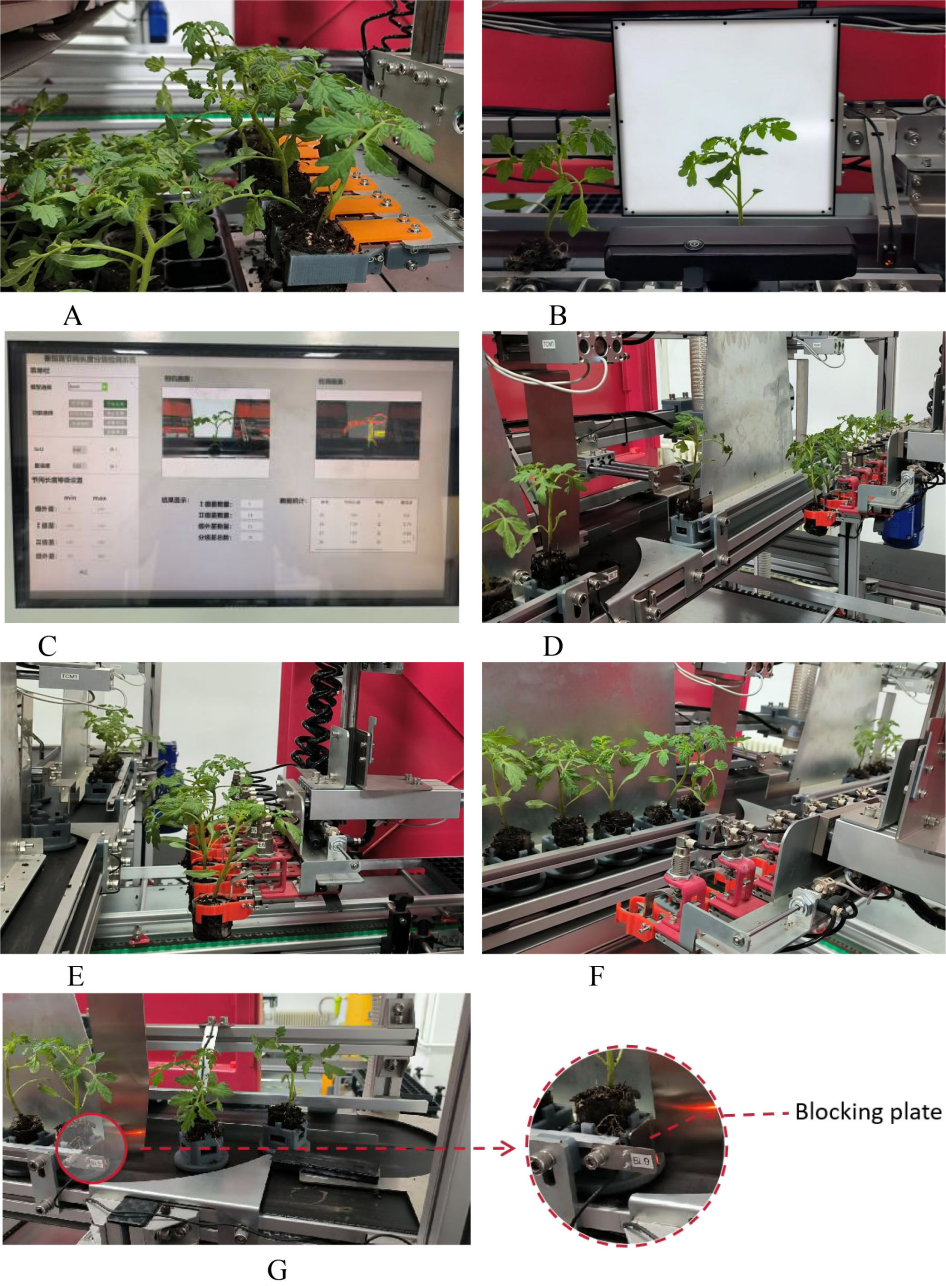
Experimental process diagram. **(A)** Seedling picking-up status. **(B)** Waiting for detection area. **(C)** Identification system detection. **(D)** Grade II seedling grabbing. **(E)** Grade I seedling grabbing. **(F)** Grading area status. **(G)** Removal area for extra seedlings.

The experimental process proceeded as follows: seedlings were picked up by a seedling supply robot (14A) -- seedlings to be graded were transported to the inspection area by a conveyor belt (14B) -- grading was performed by visual monitoring (14C) -- seedlings of the three grades were transported to their respective pick-up positions and blocked by barriers (14G). When there were 6 seedlings in each of the first and second grade pick-up positions, the first and second grade replanting robots performed the replanting operation (14DEF). Seedlings of lower grades were manually removed from their pick-up positions (14G) -- until all seedlings were graded. The experimental process is shown in **Figure 14**. For this experiment, 144 tomato seedlings were prepared. After the experiment concluded, the cumulative internode lengths of the tomato seedlings were measured manually, and it was determined whether the seedlings in each grade had been correctly classified based on their cumulative internode lengths. The aim was to verify the accuracy of DSH_YOLO in classifying the cumulative internode lengths of tomato seedlings in practical applications. During the experiment, the average inference speed reached 27 FPS, with GPU memory usage ranging from approximately 500 to 700 MB, demonstrating stable real-time performance.

**Figure 15:**
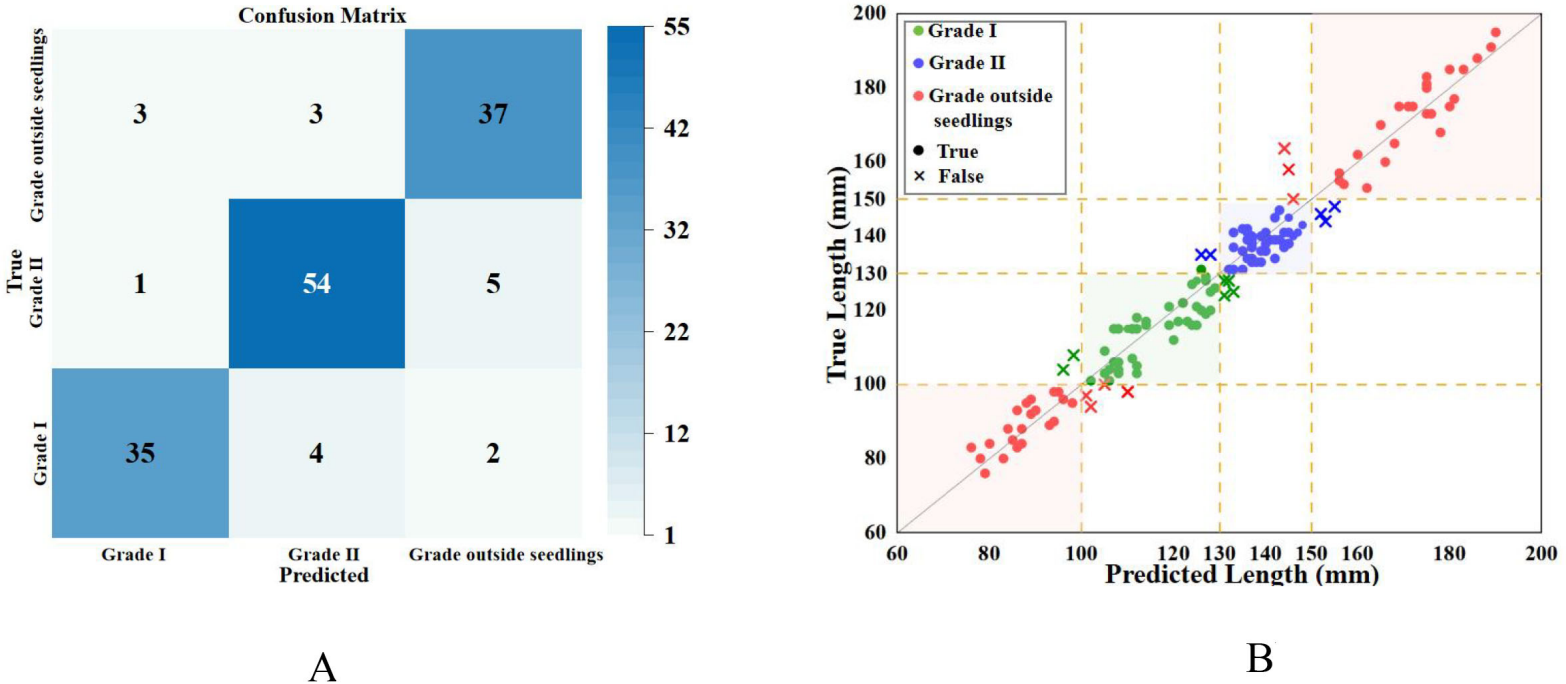
Comparison of experimental data. **(A)** Confusion Matrix. **(B)** Scatter plot.

After the experiment, the experimental data were organized into a confusion matrix as shown in **Figure 15A** and visualized as a scatter plot as shown in **Figure 15B**. The figures show that most data points fall within the range of 100-150. Furthermore, the machine’s predictions of cumulative internode length were roughly equal in both overestimation and underestimation. This is because the leaves and branches of the tomato seedlings partially obscured the nodes, preventing the model from identifying the apical meristem and the lowest cotyledon node, thus leading to an underestimation of cumulative internode length. Additionally, some tomato seedlings had fallen cotyledons, making it difficult for the model to locate them, resulting in an overestimation of length. Moreover, the data with larger measurement errors were mostly predicted data that were much smaller than manually measured data. This was likely due to the severe bending and tilting of the stems in these seedlings, hindering accurate calculation. These error issues will be key areas for improvement in future research.

The figure also shows that the classification accuracy for Grade I seedlings was 85.37%, for Grade II seedlings it was 90%, and for non-graded seedlings it was 86.05%, with an average classification success rate of 87.50%. Among these, misclassifications were more common for Grade I and unclassified seedlings. Analysis indicates that these errors occurred because, during the identification process, the smaller size of these seedlings—where leaves obscured the cotyledon nodes or stems were curved—prevented accurate identification. Grade II seedlings had the highest grading success rate. This is attributed to the fact that Grade II seedlings are taller, with stems that are typically longer and straighter, and with fewer instances of foliage obstruction, which facilitates the system’s accurate detection of cumulative internode length. Furthermore, when measuring the cumulative internode length of the 144 tomato seedlings tested, the error margin for Grade I seedlings was 8.3 mm, for Grade II seedlings 8.4 mm, and for unclassified seedlings 7.1 mm. The average error margin was only 8.0 mm—less than 1 cm—demonstrating that the detection accuracy meets practical application requirements.

## 4 Discussion

The DSH_YOLO network proposed in this study significantly improves the detection accuracy and robustness of cumulative internode length in tomato seedlings through multi-module enhancements. Ablation experiments demonstrate that the introduction of deformable convolutions effectively captures the curved morphology of the stems. The adoption of the SIoU loss function, combined with an angular penalty term to enforce alignment of the predicted bounding boxes with the stem axis, alleviates the issue of missegmentation in areas where branches and leaves intersect, resulting in a 7.2% increase in Precision. This validates the importance of directional constraints for the detection of slender structures. After further embedding a Haar wavelet downsampling module, the model significantly improved segmentation accuracy for stem edges and occluded regions through multi-frequency feature decomposition. Precision, Recall, and mAP50 increased by 11.7%, 11%, and 5.2%, respectively, indicating that preserving high-frequency texture information plays a critical role in characterizing complex morphologies. Furthermore, in prototype experiments, the correct classification rates for Grade I, Grade II, and non-graded seedlings reached 85.37%, 90%, and 86.05%, respectively, with an average classification success rate of 87.50% and an average error length of only 8.0 mm, meeting the requirements for large-scale tomato seedling grading tasks.

Compared to existing mainstream models, DSH_YOLO shows significant advantages in Precision, Recall, and mAP@0.5. However, some errors still exist in the prototype’s actual testing: for example, when the stem is bent or severely occluded, the model may miss nodes, leading to lower length predictions; for short tomato seedlings, the cumulative internode length is not obvious, making identification difficult. These issues indicate that the current model’s adaptability to extreme morphologies still has room for improvement.

The proposed tomato seedling cumulative internode length detection method can perform seedling grading tasks with high accuracy. However, the proposed algorithm DSH_YOLO still has shortcomings for achieving better performance in practical applications. First, the dataset is limited, focusing on the Millennium tomato seedling variety, which is not conducive to the promotion of cumulative internode length detection for other tomato seedling varieties; second, the algorithm struggles to perform well in extreme cases, such as when tomato seedling branches and leaves severely occlude nodes or internodes, or when the stem is severely bent. In future work, we will continue to focus on the shortcomings by optimizing the network structure, expanding the dataset to optimize the model, further improving its adaptability under multi scenario and multi crop conditions, and strengthening the integration with 3D reconstruction and other technologies to better complete the task of tomato seedling cumulative internode length detection and meet more complex precision agriculture application requirements. When applying the algorithm to graft seedling grading operations, we can further introduce indicators such as stem thickness on the basis of consistency grading in this article to improve grading standards.

## 5 Conclusion

To address the challenge of measuring the cumulative internode length of tomato seedlings, this study proposes a DSH_YOLO network based on multi-scale fusion and develops a corresponding intelligent grading system. By incorporating deformable convolutions, the SIoU loss function, and a Haar wavelet downsampling module into the YOLOv8 framework, the model’s ability to extract features from curved stems, areas obscured by nodes, and delicate internode structures has been significantly enhanced.

Based on the original YOLOv8, Deformable Convolution is introduced into the backbone to dynamically adjust the model convolution kernel to adapt to the characteristics of seedling stems. The SIoU loss function is improved to force alignment between the stem and the prediction box. A Haar wavelet downsampling module is embedded in the backbone to reduce information loss from traditional pooling operations. Ultimately, the algorithm achieves a precision of 96.1%, a recall of 94.3%, and an mAP@0.5 of 92.1%. A corresponding intelligent grading system for tomato seedlings was developed, and the algorithm was deployed on a machine for practical verification, demonstrating the feasibility of intelligent grading of tomato seedling cumulative internode length.

Prototype testing shows that the average grading success rate of the algorithm proposed in this paper reaches 87.50% across tomato seedlings of different grades, with an average length error of 8.0 mm. This demonstrates that the system can meet the requirements for large-scale grading and inspection of tomato seedlings, exhibiting high detection accuracy and success rates. It can provide ideas for grading tasks involving other types of seedlings during their growth stages and offer support for seedling production and the development of intelligent agricultural equipment.

## Data Availability

The original contributions presented in the study are included in the article/supplementary material. Further inquiries can be directed to the corresponding author.
